# Automated classification and characterization of the mitotic spindle following knockdown of a mitosis-related protein

**DOI:** 10.1186/s12859-017-1966-4

**Published:** 2017-12-28

**Authors:** Matloob Khushi, Imraan M. Dean, Erdahl T. Teber, Megan Chircop, Jonathan W. Arthur, Neftali Flores-Rodriguez

**Affiliations:** 1 Children’s Medical Research Institute, The University of Sydney, Westmead, NSW Australia; 2 Current address: School of Biomedical Sciences, The University of Queensland, St. Lucia, Brisbane, QLD 4072 Australia; 3 Current address: School of IT, The University of Sydney, Darlington, NSW Australia

**Keywords:** Image processing, Mitosis, Mitotic spindle, Automated classification, Image analysis software

## Abstract

**Background:**

Cell division (mitosis) results in the equal segregation of chromosomes between two daughter cells. The mitotic spindle plays a pivotal role in chromosome alignment and segregation during metaphase and anaphase. Structural or functional errors of this spindle can cause aneuploidy, a hallmark of many cancers. To investigate if a given protein associates with the mitotic spindle and regulates its assembly, stability, or function, fluorescence microscopy can be performed to determine if disruption of that protein induces phenotypes indicative of spindle dysfunction. Importantly, functional disruption of proteins with specific roles during mitosis can lead to cancer cell death by inducing mitotic insult. However, there is a lack of automated computational tools to detect and quantify the effects of such disruption on spindle integrity.

**Results:**

We developed the image analysis software tool MatQuantify, which detects both large-scale and subtle structural changes in the spindle or DNA and can be used to statistically compare the effects of different treatments. MatQuantify can quantify various physical properties extracted from fluorescence microscopy images, such as area, lengths of various components, perimeter, eccentricity, fractal dimension, satellite objects and orientation. It can also measure textual properties including entropy, intensities and the standard deviation of intensities. Using MatQuantify, we studied the effect of knocking down the protein clathrin heavy chain (CHC) on the mitotic spindle. We analysed 217 microscopy images of untreated metaphase cells, 172 images of metaphase cells transfected with small interfering RNAs targeting the luciferase gene (as a negative control), and 230 images of metaphase cells depleted of CHC. Using the quantified data, we trained 23 supervised machine learning classification algorithms. The Support Vector Machine learning algorithm was the most accurate method (accuracy: 85.1%; area under the curve: 0.92) for classifying a spindle image. The Kruskal-Wallis and Tukey-Kramer tests demonstrated that solidity, compactness, eccentricity, extent, mean intensity and number of satellite objects (multipolar spindles) significantly differed between CHC-depleted cells and untreated/luciferase-knockdown cells.

**Conclusion:**

MatQuantify enables automated quantitative analysis of images of mitotic spindles. Using this tool, researchers can unambiguously test if disruption of a protein-of-interest changes metaphase spindle maintenance and thereby affects mitosis.

## Background

Mitosis is a multi-step process that normally results in the equal segregation of chromosomal DNA and cytoplasmic organelles between two daughter cells. The mitotic spindle, a bipolar microtubule (MT)-based cellular structure, aligns the duplicated chromosomes at the centre of the cell during metaphase. Once correctly aligned, sister chromatids are separated and moved to opposing spindle poles during anaphase. Structural defects in the mitotic spindle can lead to the unequal segregation of chromosomes, which increases the oncogenic potential of the cell. The spindle assembly checkpoint (SAC) is a signalling protein complex that prevents this adverse situation by monitoring the proper interaction of the mitotic spindle with chromosomes. It delays the onset of anaphase until all chromosomes are stably attached to the kinetochore fibres of the spindle [1]. In addition to SAC proteins, other proteins associate with the spindle and regulate its assembly, stability and function. Moreover, many additional proteins are thought to play unknown roles in the formation and integrity of the mitotic spindle. Thus, researchers are investigating a plethora of proteins for possible unidentified mitotic roles. This might not only aid understanding of the mechanisms that regulate cell division, but also help to identify new targets via which cancer cell death can be induced with increased efficacy. MT-targeting anti-cancer therapies are currently in clinical use; however, they rarely completely eradicate neoplasms and are often hampered by issues such as mitotic slippage, resistance and toxicity. Many cells in the body do not divide or divide very rarely and thus have an extremely long cell cycle. Mitotic inhibitors would thus preferentially target cancer cells, which often divide rapidly. A high mitotic index correlates with increased malignancy. Cells are most vulnerable during mitosis. Moreover, inhibitors of mitotic intermediates have achieved promising results in clinical trials, demonstrating high selectivity and sensitivity.

Using fluorescence microscopy and other methods, our team has identified several proteins with mitotic roles. This work has sometimes involved the manual analysis of hundreds of images, which is laborious and time-consuming. Additionally, most common image analysis tools have been created for use in a range of applications and lack the sensitivity to identify and characterise specific features associated with mitotic cells. To solve this problem, we developed a novel set of algorithms, that we have called MatQuantify, for automated assessment of the effects of disruption of a given protein on the mitotic spindle.

We used MatQuantify to assess the effect of clathrin heavy chain (CHC) depletion on the architecture of the metaphase spindle. Clathrin, which plays a key role in membrane trafficking during endocytosis and exocytosis, is also important for the first stages of mitosis [2, 3]. This protein complex comprises three heavy chains and three light chains arranged in a trimer of three “legs” connected at a central vertex. During the first stages of mitosis, clathrin localises to the mitotic spindle. Transfection of small interfering RNA (siRNA) against clathrin causes defects in chromosome congression at the metaphase plane, resulting in delays in mitosis [3–5]. It has been proposed that the mitotic spindle is stabilised by a series of different types of inter-MT bridges, which are thought to span kinetochore-fibres and contribute to their stabilisation during chromosome movement. Clathrin, together with other proteins, is thought to form one type of these bridges [4, 5].

MatQuantify was used to rapidly assess 619 fluorescence microscopy images of mitotic spindles and accurately identify changes in the spindle architecture of clathrin-depleted cells.

## Methods

### Cell culture and siRNA transfection

HeLa cells were grown on glass coverslips in RPMI 1640 media (ThermoFisher Scientific) supplemented with 10% foetal bovine serum (ThermoFisher Scientific) and 1% penicillin-streptomycin (ThermoFisher Scientific) at 37 °C in 5% CO_2_. Cells were transfected with 90 nM siRNA duplexes the day after seeding using Lipofectamine 2000 (ThermoFisher Scientific) according to the manufacturer’s instructions. Fresh media was added after 6–8 h and every 12–15 h thereafter, until cells were fixed. siRNAs were purchased from SigmaAldrich and had the following sequences: luciferase, 5′-CGUACGCGGAAUACUUCGAdTdT-3′ (sense), 5′-UCGAAGUAUUCCGCGUACGdTdT-3′ (antisense) and CHC, 5′-GCAAUGAGCUGUUUGAAGA-3′, 5′-UCUUCAAACAGCUCAUUGC-3′ (antisense).

### Immunofluorescence

72 h after transfection, cells were fixed for 4 min in methanol cooled to −20 °C. Thereafter, samples were typically blocked in phosphate-buffered saline (PBS) containing 3% bovine serum albumin for 40 min at room temperature. Cells were then incubated with primary antibodies for 60–90 min, washed four times with PBS, labelled with Alexa Fluor-conjugated secondary antibodies (ThermoFisher Scientific) diluted 1:500 for 30–45 min, washed four times with PBS and mounted with ProLong Gold (Life Technologies). Cells were labelled with a mouse anti-CHC (610500, BD Biosciences) or a rabbit anti-CHC (ab21679, Abcam) antibody diluted 1:200 together with an Alexa Fluor 488-conjugated anti-α-tubulin antibody (322588, Life Technologies) and 1 μg/ml DAPI.

### Microscopy

Fixed cells were imaged using an Olympus IX70 microscope equipped for optical sectioning microscopy (DeltaVision, Applied Precision) with a 100× 1.4 NA U-Plan S-Apo objective and a CCD camera (CoolSnapHQ2, Roper Scientific). Standard filters (DAPI: 390/18, 435/48; FITC: 475/28,522/36; TRITC: 543/27, 594/45 and Cy5: 632/22, 676/34) were used. Each z series (0.3 μm interval) was acquired, deconvolved and projected using SoftWoRx (Applied Precision). The pixel intensity ranged from 0 to 65,535. Images contained 1024 × 1024 pixels.

### MatQuantify script

MatQuantify was written in MATLAB (MathWorks, USA). The source code is available from http://matquantify.sourceforge.net/. In addition, 619 RGB images of untreated, luciferase siRNA-treated and CHC-depleted cells are available. MatQuantify processes all images in the user-identified folder and writes computed measurements to a text file. Any execution errors are logged in a separate text file. The region of interest (ROI) for analysis was the mitotic spindle or DNA. Images were converted to a binary format by the Otsu thresholding method [6]. Cellular noise was removed by three strategies: i) small objects that were joined by only 1 pixel were disintegrated, ii) objects touching the border or in proximity to the border (within 3% of the total width of the image) were removed and iii) objects comprising less than 25,000 pixels were removed based on the observation that spindles and DNA are larger than this.

Additional poles, referred to as ‘satellite objects’, were also detected, which might be an additional spindle pole. Due to their small sizes and frequent detachment from the spindle body, the satellites were segmented according to the following criteria: i) a satellite object must be identified outside the initially detected spindle boundary but within 200 pixels of the centre of the spindle, ii) the extent value must be higher than 0.3 and iii) the total pixel intensity within satellites was higher than 50,000.

Where an image contained more than one ROI, each was treated as an independent object. A binary mask was prepared and used to segment spindles from the original greyscale image to perform intensity-based measurements.

### Statistical analysis

MATLAB was employed to analyse the data and to train machine learning algorithms. The normality of the quantified data was analysed visually and using the one-sample Kolmogorov-Smirnov test. The Kruskal-Wallis test was used to statistically compare the groups. Multiple testing correction was performed by the Tukey-Kramer post hoc test.

### Machine learning

Supervised machine learning is the ability of a computer to learn from example datasets and classify the test (unseen) data into the correct group. The Classification Learner App of MATLAB has 23 machine learning algorithms grouped into six classifier types: i) Decision Trees (Complex Tree, Medium Tree and Simple Tree), ii) Discriminant Analysis (Linear Discriminant and Quadratic Discriminant), iii) Support Vector Machine (SVM) (Linear SVM, Quadratic SVM, Cubic SVM, Fine Gaussian SVM, Medium Gaussian SVM and Coarse Gaussian SVM), iv) k-Nearest Neighbour Classifiers (Fine KNN, Medium KNN, Coarse KNN, Cosine KNN, Cubic KNN and Weighted KNN), v) Ensembles Classifiers (Boosted Trees, Bagged Trees, Subspace Discriminant, Subspace KNN and RUS Boosted Trees) and vi) Logistic Regression. These algorithms were trained and tested with their default settings to classify mitotic spindles.

## Results

**Fig. 1:**
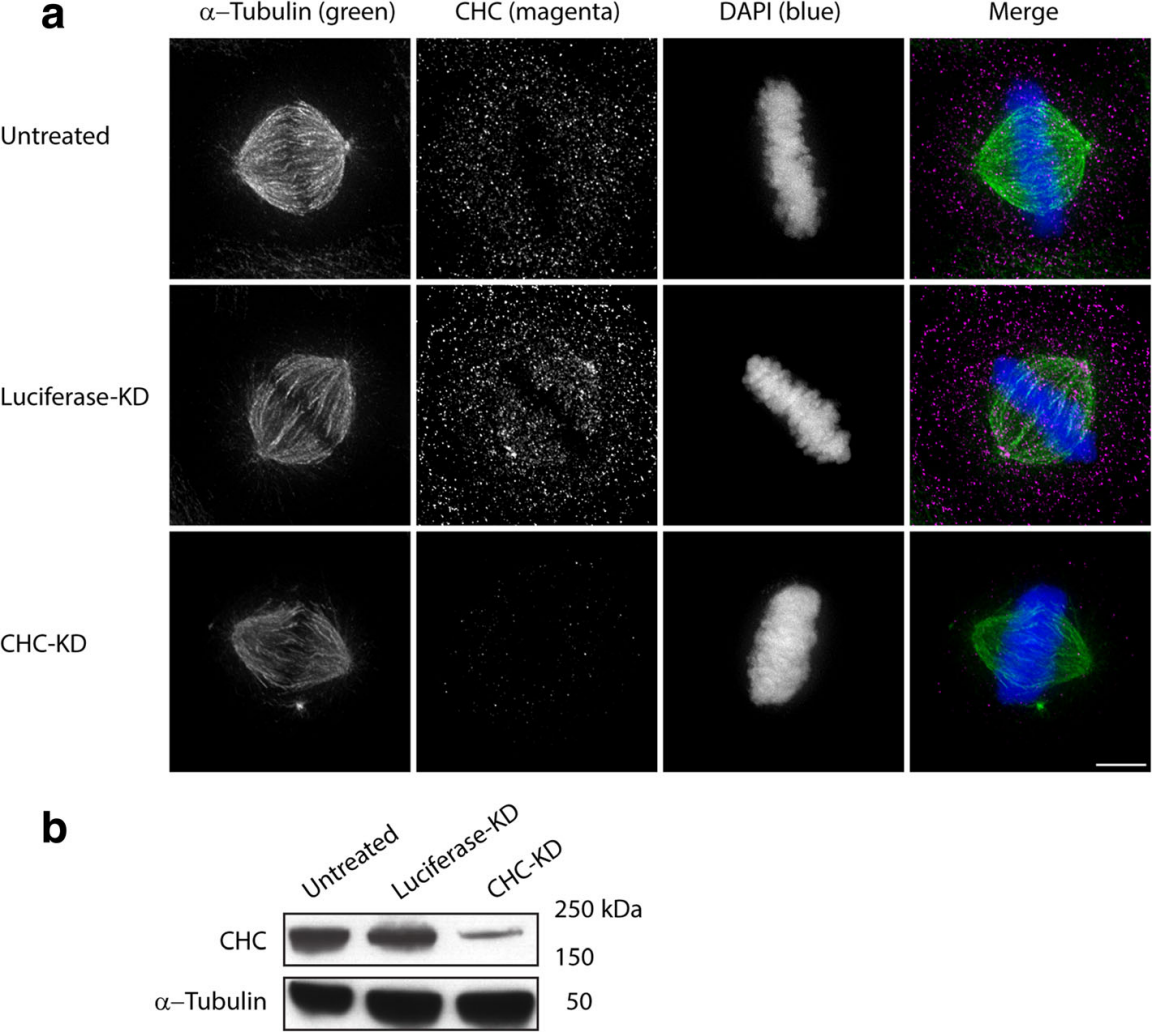
Depletion of CHC results in the formation of aberrant spindles. **a** Fluorescence microscopy images of untreated HeLa cells and those treated with luciferase-targeting siRNA (negative control) or CHC-targeting siRNA. Cells were stained with anti-α-tubulin and anti-CHC antibodies and DAPI. Scale bar, 5 μm. **b** CHC-KD in HeLa cells was assessed by western blotting. α-Tubulin was used as a loading control

**Fig. 2:**
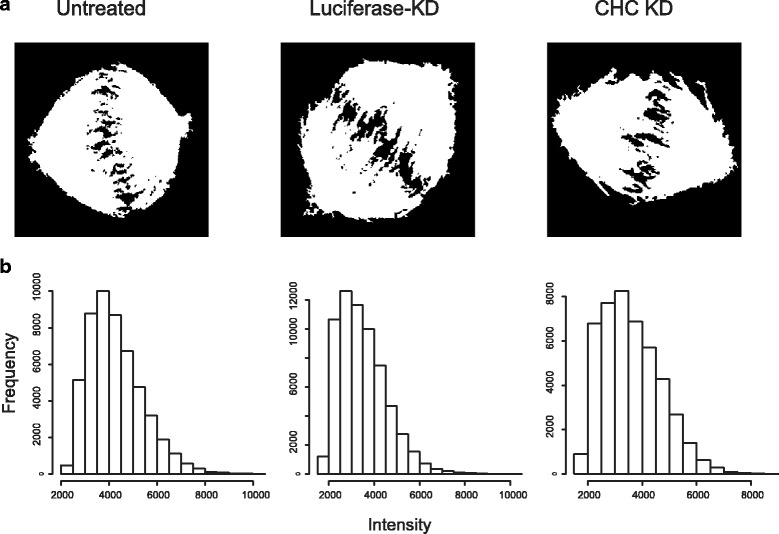
Example of binary spindle masks and intensities. **a** Binary masks of spindles shown in Fig. 1. **b** Histogram of grey-scale intensities in each of the spindles shown in **a**

CHC functions in formation of the mitotic spindle and stabilisation of kinetochore fibres [7]. Knockdown (KD) of CHC causes spindle deformation and DNA misalignment [8]. To identify the mitotic spindle properties that are associated with CHC KD, we analysed 217 images of mitotic spindles in untreated cells, 172 images of mitotic spindles in cells transfected with siRNA targeting luciferase and 230 images of CHC-depleted cells. Figure 1a shows representative mitotic spindles and DNA in these three groups, while Fig. 1b shows western blotting confirming KD of CHC. The binary masks of the spindles shown in Fig. 1a and the corresponding histograms of grey-scale intensity values within the spindles are shown in Fig. 2a and 2b, respectively. Cells transfected with luciferase-targeting siRNA served as a negative control because HeLa cells do not express this gene and thus their mitotic spindles were not expected to significantly differ from those of untreated cells. We analysed all images with an in-house-developed MATLAB script (MatQuantify) to identify measurable characteristics that differed according to whether CHC was knocked down and if these characteristics could thus be used to stratify the groups of images.

After reviewing the literature, we identified 19 properties that can be useful for defining any graphical shape, as explained in Table 1. MatQuantify saves all measurements to a tab-delimited text file. It took 5.3 min to quantify the green channel (spindle) and red channel (DNA) of 619 images using a single core of a 2.6 GHz i7 PC. The quantified data were further analysed in MATLAB.

**Table 1:** MatQuantify measures 19 properties of a segmented ROI

No.	Image property	Definition
1	Area	The number of pixels inside the region containing the ROI (mitotic spindle).
2	Convex area	The number of pixels inside the convex hull of an ROI. This is the smallest convex polygon that contains the region of interest.
3	Compactness	The degree to which a shape is compact, calculated using the formula: $Compactness=\frac{Area}{Perimeter^2}$
4	Eccentricity	An ROI can fit into an ellipse, and the roundness of the ellipse is identified by its eccentricity. The value ranges from 0 to 1. A value of 0 corresponds to a circle, while a line has an eccentricity of 1.
5	Entropy	A statistical measure of randomness characterises the texture of an image. It can be defined as: *Entropy* = − ∑ *p*. ∗ *log* 2(*p*) .* syntax means that an element in the first matrix is multiplied by the corresponding element in the second matrix.
6	Euler number	The number of objects in the region minus the number of holes in those objects, where holes are black pixels in the region of a binary image.
7	Fractal dimension	Returns the Haussdorf fractal dimension of an object represented by the binary image. Pixels with non-zero intensity belong to an object and pixels with zero intensity constitute the background.
8	Intensity (mean)	The mean intensity of all the grey-scale values in an ROI.
9	Intensity (median)	The median intensity of all the grey-scale values in an ROI.
10	Intensity (total)	The sum of all grey-scale values in an ROI.
11	Major axis length	The length (in pixels) of the major axis of the ellipse that completely encompasses the region.
12	Minor axis length	The length (in pixels) of the minor axis of the ellipse that completely encompasses the region.
13	Orientation	The angle between the x-axis and the major axis of the ellipse. The value ranges from −90° to 90°.
14	Percent density	The number of pixels that have an intensity value greater than 90% of the maximum pixel intensity value divided by the total area (in pixels).
15	Perimeter	The distance around the boundary of the region.
16	Solidity	The proportion of pixels in the convex hull that are also in the region. $Solidity=\frac{Area}{Convex Area}$
17	Standard deviation	The standard deviation of all the grey-scale values in an ROI.
18	Extent	The ratio of the total pixels in an ROI to the total pixels in the bounding box. $Extent=\frac{AreaofROI}{AreaofBoundingBox}$ The bounding box is the smallest rectangle that encompasses the region.
19	Satellites	The number of satellite objects (additional poles) identified by the algorithm.

### Classification by machine learning

The measurements obtained from the three groups of images were imported into MATLAB and combined into a table datatype. KD of luciferase is not expected to majorly affect the mitotic spindle structure; therefore, for this machine learning analysis, we labelled the quantified data from luciferase-KD cells as “untreated”. We trained 23 machine learning algorithms on quantified data of the spindle and DNA. The training data set comprised 80% of randomly selected data from both groups of images (untreated and CHC-depleted). The remaining 20% of data from each of these groups were used to test the model. In a comparison of the different algorithms, the maximum classification accuracy based on DNA was always lower than 80%, while that based on the spindle was mostly higher than 84%.

**Fig. 3:**
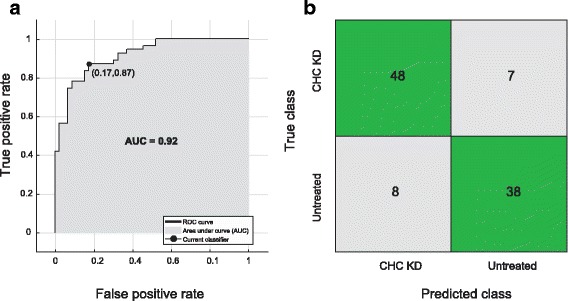
ROC plot and confusion matrix for predictions of spindle class by a SVM learning algorithm. **a** ROC plot showing the output of the used learning algorithm. SVM generated the largest Area Under the Curve (AUC) with a value of 0.92 and a true positive rate of 0.85 as marked by the point (0.17, 0.87). **b** Confusion matrix: the value shown inside the green box is derived from a true prediction while the number inside the grey box shows a false prediction

The SVM algorithm performed best in predicting the class of a randomly selected spindle image. In the receiver operating characteristic (ROC) plot, the area under the curve was 0.92 (Fig. 3a). A confusion matrix revealed that 48 of the 55 spindle images of CHC-KD cells and 38 of the 45 spindle images of untreated cells were classified correctly, meaning overall accuracy was 85.1% (Fig. 3b).

We statistically analysed the data to further understand the properties associated with each group of cells. The quantified properties did not follow a normal distribution according to the Kolmogorov-Smirnov test. Therefore, we used the non-parametric Kruskal-Wallis test followed by the Tukey-Kramer post hoc test to identify statistical differences among the three groups of cells.

### Properties of mitotic spindles in CHC-KD cells are significantly different from those in untreated and luciferase-KD cells

**Fig. 4:**
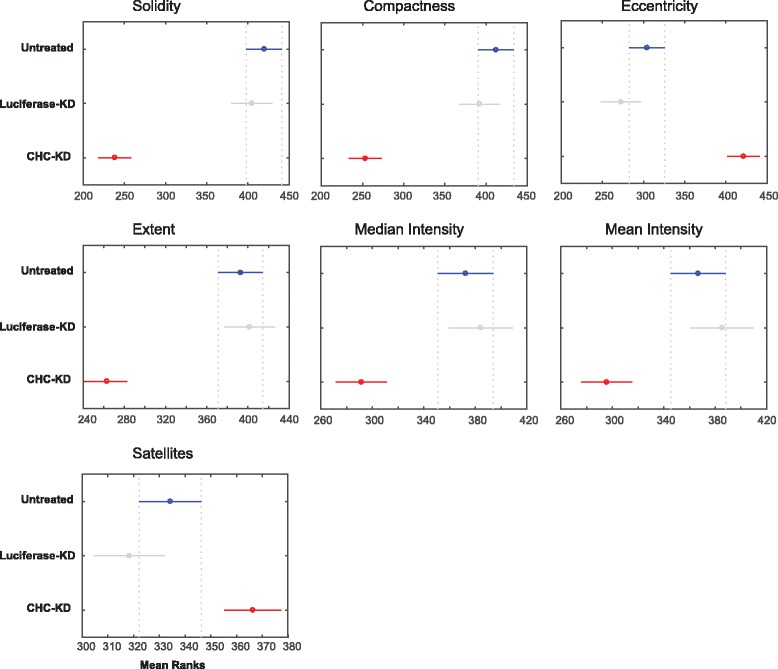
Mean ranks of CHC-KD cells significantly differ from those of untreated and luciferase-KD cells. The mean ranks of CHC-KD cells were significantly different from those of untreated and luciferase-KD cells. Ranking by the Tukey-Kramer test revealed that the comparison intervals of luciferase-KD cells overlapped with those of untreated cells, shown in grey

**Fig. 5:**
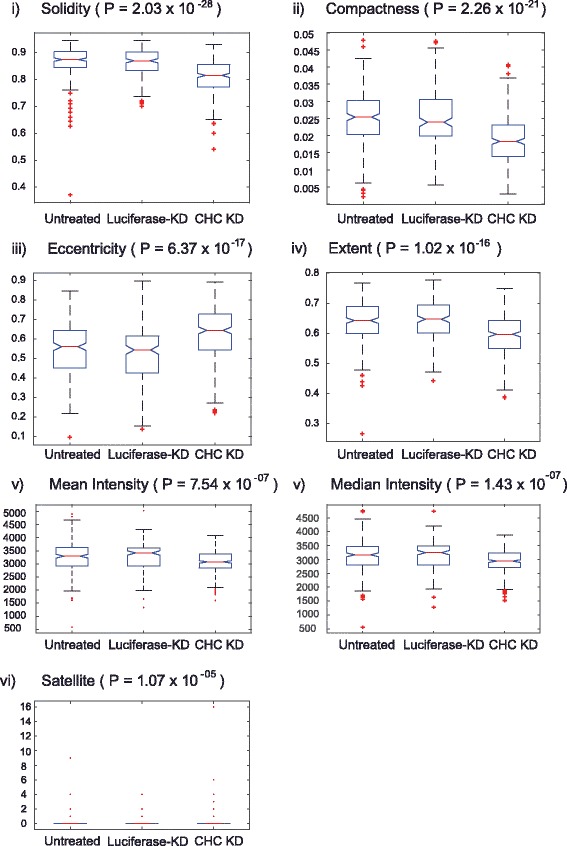
Boxplots of significantly different CHC-KD properties in comparison to untreated/luciferase-KD mitotic spindles. *P*-values were calculated using the Kruskal-Wallis test

The multiple comparison test identified that the mean ranks of solidity, compactness, eccentricity, extent, intensity (mean and median) and satellite objects quantified from untreated and luciferase-KD cells overlapped. These measurements significantly differed between CHC-KD cells and untreated/luciferase-KD cells (Fig. 4). The spread of the aforementioned measured quantities was shown as box plots (Fig. 5) and is explained below.

#### Solidity

Solidity was calculated by dividing the area of the ROI by the convex area (Table 1). This was the property that most significantly differed between CHC-KD cells and untreated/luciferase-KD cells (*P* = 2.03 × 10^−28^). Solidity was calculated from binary images. The values ranged from 0 to 1, where solidity of 1 represented a convex shape.

#### Compactness

Compactness is a function of the area and perimeter (Table 1). The significantly lower compactness of CHC-KD cells (*P* = 2.26 × 10^−21^) was associated with a larger perimeter. This relates to the rougher edges of spindles in these cells due to stray MTs.

#### Eccentricity

Eccentricity ranged from 0 (circle) to 1 (line). The eccentricity of spindles was significantly higher in CHC-KD cells than in the other two groups (*P* = 6.37 × 10^−17^). This indicates that spindles were more elliptical in CHC-KD cells and more circular in non-treated cells.

#### Extent

The extent value of spindles was significantly lower in CHC-KD cells than in luciferase-KD and untreated cells (*P* = 1.02 × 10^−16^). Extent was calculated by dividing the area of the ROI by the area of a bounding box, which was the smallest rectangle that completely encompassed the ROI. This indicates that spindles were more rectangular in untreated and luciferase-KD cells than in CHC-KD cells.

#### Intensity (mean and median)

The means and medians of fluorescence intensities within the ROIs (spindle or DNA) were calculated. The mean (*P* = 7.54 × 10^−07^) and median (*P* = 1.43 × 10^−07^) intensities were significantly lower in CHC-KD cells than in luciferase-KD and untreated cells. This confirms the finding of Giladi et al. [9] that the fluorescence intensity is reduced in compromised mitotic spindles under the influence of an electric field.

#### Satellite objects

Additional poles surrounding the spindle were counted. The number of spindles with additional poles was significantly higher in CHC-KD cells than in untreated and luciferase-KD cells (*P* = 1.07 × 10^−05^).

### Other measured properties

**Fig. 6:**
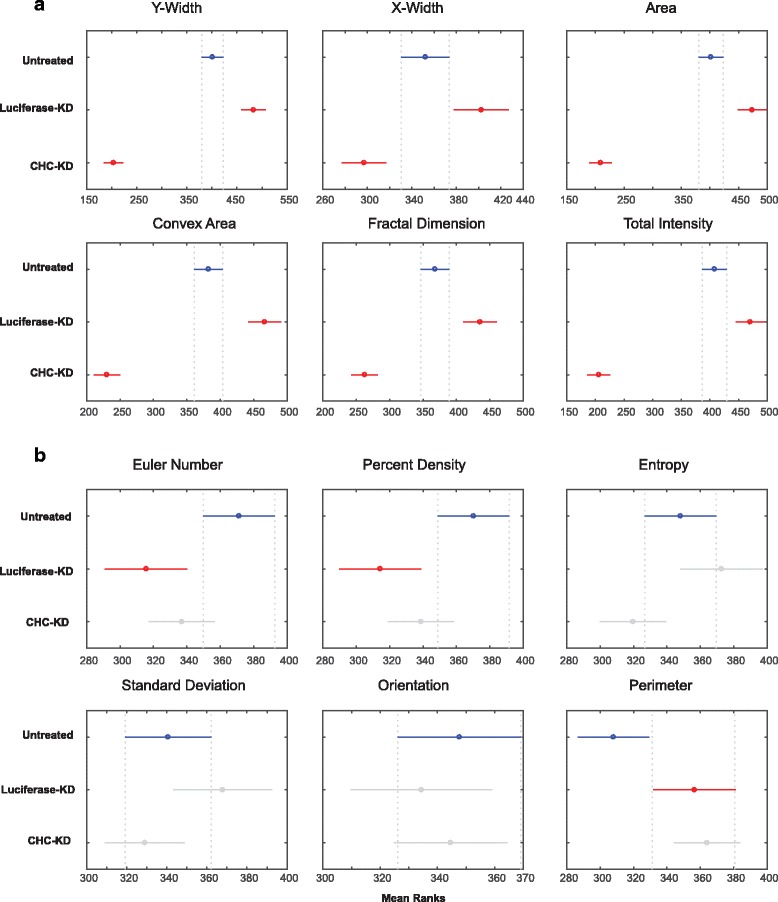
Ranking of other (non)overlapping comparison intervals. **a** Ranking of the groups where comparison intervals did not overlap. **b** Ranking of the CHC-KD group where in comparison intervals overlap with those from either the untreated or luciferase-KD cells or with the comparison intervals from these both two control groups

We did not expect luciferase-KD to affect the spindle characteristics. However, multiple comparison testing revealed that the mean ranks of widths, area, convex area, fractal dimension and total intensity significantly differed between the three groups (Fig. 6a). The comparison intervals for Euler number, percent density, entropy, standard deviation, spindle orientation and perimeter measurements of CHC-KD cells overlapped with those of untreated and/or luciferase-KD cells (Fig. 6b). These measurements can be useful for other types of image analyses [10]. Some of these measured properties are easy to understand, such as area, perimeter and axis widths. We will explain a few of the more complex measurements in more detail.

#### Fractal dimension

The complexity of shape is measured by the fractal dimension. We employed the fractal dimension algorithm sourced from the MATLAB file exchange [11, 12]. The higher the fractal dimension, the more complex an image is said to be. Our statistical analysis (Kruskal-Wallis test followed by the Tukey-Kramer post hoc test to correct for multiple testing) revealed that fractal dimension significantly differed (*P* = 8.39 × 10^−20^) between the three groups (Fig. 6). Many studies have found that fractal dimension is an important indicator to define shape [10]. Using the Mann-Whitney test, we also confirmed that fractal dimension significantly differed (*P* < 0.0001) between untreated and CHC-KD cells.

#### Percent density

The percent density is measured by dividing the number of pixels above a certain intensity threshold by the total number of pixels in the ROI. We set this threshold to 90% of the maximum greyscale pixel intensity value. This property was not significantly associated with CHC-KD spindles, because the comparison interval of CHC-KD cells overlapped with those of untreated (Fig. 6b). However, others have successfully used this metric to stratify images [13].

## Discussion

Analysis of mitotic spindle shape and structure is important for investigating spindle defects and dynamics. However, it is a laborious, time-intensive and potentially error-prone task when conducted manually. MatQuantify segments an ROI (in this case, the mitotic spindle) and measures 19 characteristics to elucidate its important properties. We anticipate that if a treatment visibly affects an ROI, quantified data obtained using MatQuantify can be used to characterise the differences between untreated and treated cells.

We studied the effect of knocking down a mitosis-related protein, CHC, on the structure of the mitotic spindle. Six properties of the mitotic spindle significantly differed between CHC-KD and untreated/luciferase-KD cells. Five of these six properties (solidity, extent, eccentricity, compactness and satellite objects) were related to structural deformation of the spindle. Fluorescence intensity, a non-structural property, was also significantly reduced in compromised spindles, in line with a previous study [9]. Our study showed that image processing with MatQuantify can identify structural changes in the mitotic spindle induced by knocking down the mitosis-related protein CHC.

All images were deconvolved after microscopy acquisition using the softWoRx tool. Deconvolution corrects for blur, noise, scatter and glare. Therefore, we did not de-noise pixel intensities within ROIs before calculating intensity-related measurements. However, further denoising can be achieved using median or Weiner filters, as we showed in another biological application [14]. We did not use absolute intensity values to calculate the image properties in Table 1, except for counting satellites. Thus, users of MatQuantify might need to adjust this parameter according to their experimental conditions. The spectral characteristics, such as the lens magnification and filters, and the collection efficiency of the fluorescence microscope used, as well as the dye microenvironment and label density, affect the absolute fluorescence intensity.

We employed the Kruskal-Wallis and Tukey-Kramer post hoc tests instead of non-parametric t-tests, which can be used to identify pair-wise statistical differences between two groups. Our study included three groups, while t-tests are only designed to compare two groups. This can lead to misinterpretation of the data. For example, using the Mann-Whitney test, which compares ranks and cumulative distributions between two groups, area and fractal dimension were found to significantly differ (*P* < 0.0001) in pairwise comparisons between the three groups. However, employing Kruskal-Wallis test following by the Tukey-Kramer test showed that area and fractal dimensions of CHC-KD group were not significantly different from untreated/luciferase-KD groups. Thus, this approach provides a better interpretation of the data.

During image processing, we removed noise from the surrounding area. However, this noise may have arisen from important cellular structures in the immediate vicinity of the spindle apparatus. A MT-independent mechanism underlies the accumulation of important proteins in the spindle envelope. Therefore, better visualisation techniques are needed to analyse the crowded region surrounding the spindle [15] and we expect to be able to develop generic image processing tools such as MatQuantify to analyse these structures.

We have shown that through machine learning algorithms, the quantified data generated by MatQuantify can be used to automatically classify an image into the ‘treated’ or ‘untreated’ group. We achieved 85% accuracy using SVM, which we considered sufficient to demonstrate the working of the method. The default parameters of SVM worked well and the accuracy was not improved by modifying the parameters. In the future, we aim to improve the accuracy by combining the quantified DNA and spindle data.

Computer algorithms have been used to assess certain aspects of the spindle structure such as orientation [16] and MT dynamics [17]. However, there is no general tool that can quantify changes in the spindle structure and other cellular structures such as DNA. MatQuantify segments an ROI based on its area and can therefore be used to measure 19 structural properties of any organelle at any magnification. The output is saved into a tab-delimited text file, which can be imported into a database for large-scale analysis [18]. The user needs to know the size of their structure-of-interest, which can be worked out by trial and error.

## Conclusions

In summary, MatQuantify measures a number of properties of an ROI and enables investigators to rapidly analyse fluorescence microscopy images in a high-throughput and automated fashion. These measurements can then be used in a machine learning approach to classify images on the basis of perturbations to the mitotic spindle, in this case due to KD of CHC. MatQuantify, and the classification method in the study should be applicable to other situations, such as pharmacological interventions, electrical fields and external radiation therapies that impact the shape and structure of the mitotic spindle.

## Abbreviations

CHC: Clathrin heavy chain
KD: Knockdown
MT: Microtubule
PBS: Phosphate-buffered saline
ROC: Receiver operating characteristic
ROI: Region of interest
SAC: Spindle assembly checkpoint
siRNA: Small interfering RNA
SVM: Support Vector Machine
